# Effect of vitamin D supplementation on inflammation and nuclear factor *kappa*-B activity in overweight/obese adults: a randomized placebo-controlled trial

**DOI:** 10.1038/s41598-017-15264-1

**Published:** 2017-11-09

**Authors:** Aya Mousa, Negar Naderpoor, Josphin Johnson, Karly Sourris, Maximilian P. J. de Courten, Kirsty Wilson, Robert Scragg, Magdalena Plebanski, Barbora de Courten

**Affiliations:** 1 Monash University, Monash Centre for Health Research and Implementation (MCHRI), Melbourne, VIC 3168 Australia; 2 Baker IDI Heart and Diabetes Institute, Melbourne, VIC 3004 Australia; 3 Victoria University, Centre for Chronic Disease, Melbourne, VIC 3021 Australia; 4 Monash University, Department of Immunology and Pathology, Melbourne, VIC 3004 Australia; 5 The University of Auckland, School of Population Health, Auckland, 1072 New Zealand

## Abstract

*In-vitro* studies suggest that vitamin D reduces inflammation by inhibiting nuclear factor kappa-B (NFκB) activity. Yet, no trials have examined the effects of vitamin D supplementation on NFκB activity *in-vivo* in humans. We conducted a double-blind randomized trial (RCT) examining effects of vitamin D supplementation on inflammatory markers and NFκB activity in peripheral blood mononuclear cells (PBMCs). Sixty-five overweight/obese, vitamin D-deficient (25-hydroxyvitamin D [25(OH)D] ≤ 50 nmol/L) adults were randomized to a single 100,000 IU bolus followed by 4,000 IU daily cholecalciferol or matching placebo for 16 weeks. We measured BMI, % body fat, serum 25(OH)D, high-sensitivity C-reactive protein (hsCRP), tumour necrosis factor (TNF), monocyte chemoattractant protein-1 (MCP-1), interferon-gamma (IFN-γ), several interleukins, and NFκB activity in PBMCs. Fifty-four participants completed the study. Serum 25(OH)D concentrations increased with vitamin D supplementation compared to placebo (p < 0.001). Vitamin D and placebo groups did not differ in any inflammatory markers or NFκB activity (all p > 0.05). Results remained non-significant after adjustment for age, sex, and % body fat, and after further adjustment for sun exposure, physical activity, and dietary vitamin D intake. Although *in-vitro* studies report anti-inflammatory effects of vitamin D, our RCT data show no effect of vitamin D supplementation on inflammatory markers or NFκB activity *in-vivo* in humans.

## Introduction

Chronic low-grade inflammation is common in obesity and plays an important role in the etiology of many chronic diseases including type 2 diabetes and cardiovascular disease^1^. While reducing obesity is effective in reducing inflammation and delaying disease onset and progression, weight-loss strategies on a population scale are difficult to achieve and maintain in the long-term^1^. Identification of additional easily modifiable risk factors is therefore needed to mitigate risk factors associated with chronic diseases, including inflammation.

Increasing evidence suggests that vitamin D may have a role in inflammation and immunoregulation^2^. Yet, vitamin D deficiency remains prevalent worldwide^3^. Most experts define vitamin D deficiency as 25-hydroxyvitamin D (25(OH)D) concentrations ≤ 50 nmol/L; however optimal concentrations required for extra-skeletal health are debated^3^. Nevertheless, it is of concern that 20–60% and 10–40% of the UK and US populations, respectively, have vitamin D levels < 50 nmol/L^4^. An adequate vitamin D status is achieved primarily via cutaneous synthesis upon exposure to ultraviolet (UV) B radiation, and can also be derived from diet and supplements^5^. However, the rising prevalence of obesity, sedentary indoor lifestyles, and current sun exposure restrictions to prevent skin cancer have made it difficult to obtain adequate vitamin D through sun exposure, and few foods are naturally high in vitamin D or vitamin D fortified^6^.

A role for vitamin D in influencing inflammatory and immune responses is supported by the presence of the nuclear vitamin D receptor (VDR) in most immune cells including monocytes, macrophages, and activated T and B lymphocytes [10]. *In vitro* studies report that active vitamin D (1,25-dihydroxyvitamin D_3_ [1,25(OH)_2_D_3_]) inhibits pro-inflammatory cytokine expression, promotes anti-inflammatory cytokine production, and regulates immune cell activity^7–9^. These effects are believed to occur partly via inhibition of the nuclear factor *kappa-*B (NFκB) pathway^10–14^, a transcription factor with a critical role in inflammatory and immune responses, as well as in the pathophysiology of several chronic diseases such as cancer, asthma, and diabetes^15,16^. *In vivo* administration of 1,25(OH) _2_D_3_ in mice has been shown to modulate chemokine and cytokine expression and to prevent the development of inflammatory arthritis, type 1 diabetes, and autoimmune encephalomyelitis^17^. Animal models also show that treatment with 1,25(OH)_2_D_3_ reduces NFκB signaling by increasing VDR expression^18,19^.

Human studies are limited. To the best of our knowledge, no previous observational studies or randomized trials have examined the effect of vitamin D on NFκB activity *in vivo* in humans. For inflammatory markers, some^20–23^, but not all^24,25^, cross-sectional studies have reported inverse associations between 25(OH)D concentrations and C-reactive protein (CRP), tumor necrosis factor (TNF), and interleukin-6 (IL-6) in healthy^21–23^ or overweight/obese populations^20^. However, few good-quality randomized controlled trials (RCTs) have examined the effects of vitamin D supplementation on inflammatory markers. A recent systematic review of RCTs in overweight and obese individuals highlighted important limitations in existing trials including low supplementation doses, variability in participants’ vitamin D status, lack of assessment of lifestyle factors (diet, exercise, sun exposure), and the use of co-interventions including calcium and/or weight reduction programs in most trials^26^. These limitations have made it difficult to interpret findings.

We aimed to address these limitations by examining the effects of vitamin D supplementation on inflammatory markers and NFκB activity in overweight or obese but otherwise healthy individuals, with vitamin D-deficiency (25(OH)D ≤ 50 nmol/L), who receive a sufficient dose (≥4000 IU daily) of supplementation, in a clinical trial without co-interventions and with rigorous methods to minimize confounding by lifestyle factors. We hypothesized that vitamin D supplementation would decrease pro-inflammatory markers and NFκB activity and increase anti-inflammatory markers, compared with placebo.

## Methods

### Study Design and Participants

This study was part of a parallel-group, double-blind, randomized, placebo-controlled trial designed to assess the effects of vitamin D supplementation on insulin sensitivity and secretion, and a detailed trial protocol is published^27^. Briefly, 65 overweight/obese but otherwise healthy adults were recruited over a two-year period from the local community in Melbourne, Australia, via posters, flyers, email newsletters and online social media and community websites. Participants were screened and those with serum 25(OH)D concentrations ≤ 50 nmol/L on screening were recruited if they met the following inclusion criteria: aged 18–60 years, generally healthy on medical screening, overweight or obese (body mass index (BMI) ≥ 25 kg/m^2^; weight < 159 kg due to facility restrictions), with a stable weight (<5 kg change in the preceding year) and no intention to lose weight or change their diet or physical activity for the trial duration. Exclusion criteria included: smoking or high alcohol use (>4 and >2 standard drinks/week for men and women, respectively); use of medication, vitamins, or supplements; hypercalcemia or allergies; diabetes (previously diagnosed or based on oral glucose tolerance test [OGTT]) or any major diseases including active cancer in the preceding 5 years; or presence of acute inflammation (based on medical history or physical or laboratory examination). Women who were pregnant, lactating, or experiencing menopause were excluded.

### Ethics

All participants provided written informed consent prior to commencing the trial. The trial was conducted according to the principles of the Declaration of Helsinki^28^ and received ethical approval from the Monash University Human Research Ethics Committee and Monash Health (Protocol ID: CF13/3874-2013001988). The trial was registered on clinicaltrials.gov (ID: NCT02112721) on April 10^th^, 2014.

### Intervention and Randomization

Participants were randomized to either the vitamin D group receiving an initial bolus dose of 100,000 IU (in 2 capsules) followed by 4,000 IU (in 4 capsules) of cholecalciferol daily, or the placebo group receiving an equivalent number of identical placebo capsules, which were continued daily for a period of 16 weeks. The bolus dose selected is well below levels associated with toxicity or adverse effects^29^. All participants were instructed to consume the four capsules daily, and maintain their usual diet and exercise habits.

Randomization was performed as described previously^27^. Briefly, randomization was performed by an independent researcher not involved in the trial using a computerized random sequence generation program. Participants were randomized in blocks of four by sex and time of study entry (seasons) to ensure balance between sexes in each test group and to control for the effect of seasonal change. All capsules were identical and tasteless to maintain blinding, and all participants, investigators, and outcome assessors remained blinded until data analysis was complete. Compliance was assessed by the empty pill containers returned by participants at the final follow up visit and by post-intervention 25(OH)D concentrations.

### Data Collection and Analyses

Detailed descriptions of data collection and outcome measures are reported in our published protocol^27^. Outcome measures were obtained at baseline prior to the initial bolus of vitamin D and repeated after 16 weeks of supplementation. Participants who were eligible on phone screening attended a medical screening which included medical history, physical examination (including anthropometry, and pregnancy tests for women), measurement of serum 25(OH)D concentrations to document vitamin D status, and an OGTT to exclude diabetes according to World Health Organization guidelines^30^.

#### Anthropometric and Lifestyle Assessments

Body composition was measured by dual X-ray absorptiometry (DEXA) (Monash Health Radiology, Melbourne). Body mass index (BMI) was calculated from weight (kg)/square of height (m), and waist and hip circumferences were used to establish the waist-to-hip ratio (WHR) as an additional index for body fat distribution (waist (mm)/hip (mm) = WHR). Participants completed validated questionnaires assessing self-reported sun exposure habits, physical activity (International Physical Activity Questionnaire)^31^, and diet (3-day food record; Foodworks 8.0 Professional, Xyris Software, Australia).

#### Blood Sample Analyses

Fasting venous blood samples were drawn at baseline, and then after the 16-week intervention using standard phlebotomy techniques into serum separation vacutainers for measurement of serum inflammatory markers and 25(OH)D concentrations, respectively. All blood samples were analyzed under blinded conditions using standard quality control systems (all results within ± 2 SD). Serum 25(OH)D and hsCRP were measured by an accredited and quality-assured laboratory (Monash Health Pathology, Melbourne). Serum 25(OH)D was measured using direct competitive chemiluminescent immunoassays (CLIA) (DiaSorin Inc., MN, USA) with inter- and intra-assay coefficients of variation (CVs) of <10% and <4%, respectively. Plasma high sensitivity CRP (hsCRP) was measured by highly sensitive near-infrared particle immunoassay rate methodology on a Synchron LX system analyzer (Beckman Coulter Inc., Sydney, Australia) according to manufacturer’s instructions. Inter- and intra-assay CVs for hsCRP were <3% and <5%, respectively. Inflammatory markers were measured using a bead-based multi-analyte assay (LEGENDplex™ Human Inflammation panel, Cat. 740118; Biolegend, San Diego, CA). This panel was used to simultaneously quantify 13 pro- and anti- inflammatory cytokines/chemokines including TNF, monocyte chemoattractant protein-1 (MCP-1), interferon alpha (IFN-α) and gamma (IFN- γ), and IL-1β, IL-6, IL-8, IL-10, IL-12, IL-17A, IL-18, IL-23, and IL-33. Serum samples were diluted 2-fold based on preliminary testing and the kit-specified protocol was performed on 96-well v-bottom plates as follows. Sonicated pre-mixed beads, serum samples, cytokine standards, and detection antibodies were added to each well and then plates were incubated (in the dark) on a plate shaker at 600 rpm for 2 hours at room temperature (RT). Following incubation, streptavidin–phycoerythrin (SA-PE) solution was added to each well and further incubated on a plate shaker for 30 min at RT. The conjugated beads were then centrifuged (1000 *g*, 5 minutes, RT) and pelleted, and resuspended in wash buffer before being transferred to mini FACS tubes for data acquisition. Data acquisition was performed using the BD™ LSR II flow cytometer and FACS DIVA software set up as per manufacturer’s instructions (Becton Dickinson, San Diego, CA). Data were analyzed using the LEGENDplex™ Data Analysis Software (BioLegend, San Diego, CA) with standard curves generated from 0 to 50,000 pg/ml, and samples adjusted for the dilution factors. Inter- and intra-assay CVs for all analytes were <8% and <9%, respectively. Of the 13 cytokines, two were undetectable (IFN-γ and IL17A) in most of the samples and were therefore excluded from the analysis.

#### Measurement of NFκB activity

Peripheral blood mononuclear cells (PBMCs) were isolated by centrifuging fasting whole blood samples collected in BD Vacutainer® CPT™ cell preparation tubes with sodium citrate. The harvested PBMC pellet was resuspended in fetal bovine serum (FBS) with 10% dimethylsulphoxide (DMSO) and stored at −80 °C. Immediately prior to analysis, PBMC samples were thawed and washed in excess phosphate buffered saline (PBS) and centrifuged (400 *g*, 5 minutes, 4 °C) to remove residual FBS and DMSO. The supernatant was discarded and the pellets resuspended in 100 μL of triple detergent buffer. These were then sonicated for 1 minute and then centrifuged (400* g*, 3 minutes, 4 °C). Resulting supernatant was used in the NFκB assay. Protein concentration of all protein isolates was determined using the Pierce Bicinchoninic Acid protein assay (Pierce, Rochford, IL), performed according to manufacturer’s instructions. The TransAM NFκB DNA-binding activity assay (Active Motif, Carlsbad, CA) was used to detect and quantify NFκB transcription factor activation, specifically of the p65 subunit. Nuclear extracts obtained from PBMC samples (30 μg protein per well) were analyzed for their binding capacity to an NFκB consensus sequence in labelled DNA in a 96 well plate format. The assays were performed according to manufacturer’s instructions and absorbance was measured on a Victor3V Multilabel plate reader (Perkin Elmer, Wellesley, MA). Results are expressed as pg p65 activity per μg protein (pg/μg protein).

### Statistical Analyses

The sample size calculation has been reported elsewhere^27^, and was based on the main study where insulin sensitivity was the primary outcome. Analyses were performed on a per-protocol basis using Stata V.12.1 (StatCorp LP, USA). Shapiro-Wilk tests and visual inspection of histograms and scatterplots were used to assess normality with the assistance of an experienced biostatistician. Baseline characteristics are presented as mean ± SD and frequencies (%), or median (interquartile range [IQR]) for skewed variables. Due to the skewed distributions of the inflammatory markers and NFκB activity, all results were analyzed using non-parametric tests. Spearman correlations and Mann-Whitney U-tests were used to examine associations at baseline between 25(OH)D concentrations and continuous and categorical variables, respectively. Within-group differences were assessed using Wilcoxon signed-rank tests. Differences between treatments groups were assessed using Mann-Whitney U-tests and chi-squared tests for continuous and categorical variables, respectively. Efficacy of the intervention on the outcomes (between-group differences) were analyzed using changes in outcome variables and quantile multiple regression (to examine difference in medians). In quantile multiple regression, we adjusted for those variables which were significantly associated with the outcome measure based on Spearman correlations, as well as other clinically relevant variables including baseline values, age, sex, and % body fat, and factors which may affect vitamin D status including season, sun exposure, and dietary vitamin D intake. Pre-specified subgroups including those with baseline 25(OH)D ≤ 30 nmol/L were assessed as well as obese (BMI ≥ 30 kg/m^2^) subgroups. Further exploratory analyses were conducted in subgroups of participants with high baseline concentrations (≥median values) of selected inflammatory markers. All tests were two-sided and Bonferroni correction was used to adjust for multiple testing *(p* = *0.05*/*total number of tests (n* = *13)* = *0.004)*. Statistical significance was therefore set at p < 0.004.

### Data Availability

The datasets generated and analysed during the current study are available from the corresponding author on reasonable request.

## Results

### Sample and baseline characteristics

**Figure 1:**
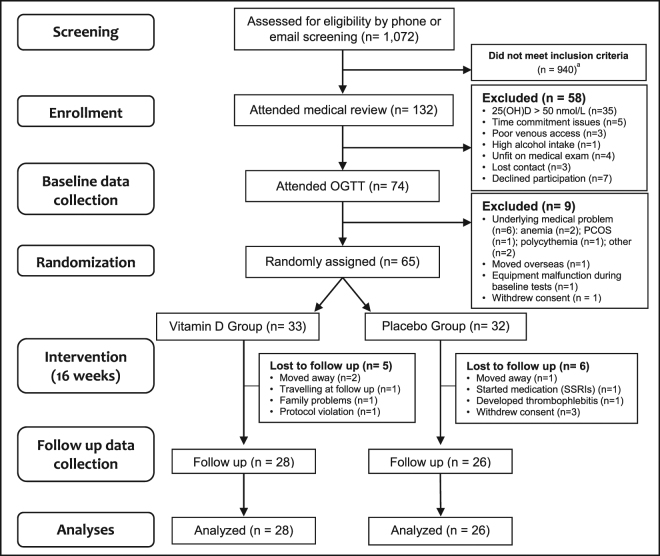
Participant Flowchart: Numbers of participants who were recruited, randomized, dropped out, and analyzed during the trial. ^a^Majority of interested participants did not meet criteria due to taking medication/supplements; not being overweight/obese; or not being interested after receiving a detailed description of study procedures. Abbreviations: 25(OH)D, 25-hydroxyvitamin D; OGTT, oral glucose tolerance test; PCOS, polycystic ovary syndrome; SSRIs, selective serotonin reuptake inhibitors.

The participant flowchart is presented in Fig. 1. Of 1,072 participants screened for eligibility, 132 attended medical screening and 65 were successfully randomized between September 2014 and July 2016 (33 and 32 in the vitamin D and placebo groups, respectively) (Fig. 1). By the end of the study, nine participants had dropped out and two were withdrawn (one due to protocol violation and one due to an adverse event of thrombophlebitis after an intravenous glucose tolerance test). The remaining 54 participants (vitamin D group = 28 and placebo group = 26) completed the study and were analyzed in a blinded per-protocol fashion. Baseline demographic, anthropometric, and biochemical characteristics of both groups are presented in Table 1. Baseline characteristics did not differ between drop outs and non-drop outs (all p > 0.05) (data not shown).

**Table 1:** Participant demographics and baseline characteristics.

Characteristics	Vitamin D group (*n* = 28)	Placebo group (*n* = 26)	*p*
Male, *n* (%)	17 (60.7)	18 (69.2)	0.5
Age (years)	30.5 (25–35)	29.5 (25–41)	0.9
Ethnicity^a^, *n*			
Caucasian	9	5	0.2
South and Central Asian	8	9	
South-East and North-East Asian	4	9	
Other^b^	5	2	
Sun exposure (index score)^c^	4.2 (1.6–6.3)	5.1 (2.0–6.9)	0.5
Physical activity (IPAQ-METS score)^c^	1751 (920–3510)	2912 (1485–5544)	0.2
Dietary vitamin D intake (IU)^c^	91.1 (54.6–130.9)	73.1 (61.0–110.8)	0.3
Season of blood collection, *n* (%)			
Winter	5 (17.8)	8 (30.7)	0.3
Spring	11 (39.3)	4 (15.4)	
Summer	8 (28.6)	10 (38.5)	
Autumn	4 (14.3)	4 (15.4)	

Data are expressed as frequency (n, %) or median (interquartile range), unless otherwise specified.

*p* = Mann-Whitney U-tests and chi-squared tests for baseline differences between treatment groups; ^a^Ethnicity was determined by self-report (n = 51/54 reported ethnicity); ^b^refers to African, Middle-Eastern, South-American, and Polynesian ethnicities; ^c^calculated from self-reported questionnaires and food records as previously reported^27^; Abbreviations: IPAQ-METS, international physical activity questionnaire- multiples of the resting metabolic rate.

Thirty-five male and 19 female participants aged 30 (25–36) years (median [IQR]) with a BMI of 30.1 (27.7–33.2) kg/m^2^, and % body fat of 39.6 ± 8.7% (mean ± SD) completed the study. Mean baseline serum 25(OH)D concentration was 32.7 ± 11.4 nmol/L (range = 9–50 nmol/L), with 43% of participants (n = 23) having a 25(OH)D concentration ≤ 30 nmol/L. Demographic, anthropometric and biochemical measures did not differ between the treatment groups at baseline, nor did physical activity, dietary intake of vitamin D (Table 1), or diet composition comprising of total energy, protein, fiber, fat, and carbohydrate intake (data not shown). Baseline 25(OH)D concentrations were not associated with age or anthropometric measures or with baseline values or change scores for any of the outcome measures (all p > 0.1; data not shown). CRP levels at baseline were positively associated with total % body fat (r = 0.40, p = 0.002) and fat mass (r = 0.30, p = 0.03) and both CRP and IL-33 were negatively associated with fat-free mass (r = −0.33, p = 0.01 and r = −0.27, p = 0.04, respectively). Patient characteristics including age and anthropometric variables were not associated with other inflammatory markers or NFκB activity at baseline (all p > 0.05; data not shown). NFκB activity at baseline was inversely associated with baseline levels of TNF, IL10, IL-1β, IL-8, IL-12, IL-23, and IL-33 (all p < 0.05; data not shown).

### Changes in serum 25(OH)D concentrations

As previously reported^32^, mean serum 25(OH)D concentration after the 16-week intervention was 63.2 ± 32.1 (range = 9–147 nmol/L). Serum 25(OH)D concentrations increased significantly in the vitamin D group, from 31.4 ± 12.6 at baseline to 88.4 ± 21.0 nmol/L at 16 weeks (p < 0.001), with no change in the placebo group for the same time-period (34.2 ± 10.0 and 36.1 ± 15.3 nmol/L, respectively; p = 0.5) (Table 2). All but one participant in the vitamin D group achieved a 25(OH)D concentration ≥ 60 nmol/L, with 82% (n = 23/28) and 64% (n = 18/28) having 25(OH)D concentrations >70 and >75 nmol/L at follow-up, respectively.

**Table 2:** Comparison of outcomes before and after 16 weeks of supplementation in both groups.

Outcome Variable	Vitamin D Group (n = 28)				Placebo Group (n = 26)				*p*_3_	*p*_4_
	Baseline	Follow Up	*p*_1_	Change	Baseline	Follow Up	*p*_2_	Change		
25(OH)D (nmol/L)	31.4 ± 12.6	88.4 ± 21.0	<0.001	57.0 ± 21.3	34.2 ± 10.0	36.1 ± 15.3	0.5	1.9 ± 15.1	0.4	<0.001
BMI (kg/m^2^)	30.2 (28.4, 34.5)	30.4 (28.7, 34.5)	0.9	0.003 ± 0.9	29.8 (27.6, 32.6)	29.2 (27.5, 32.6)	0.5	−0.1 ± 1.2	0.4	0.7
WHR	0.94 ± 0.08	0.93 ± 0.07	0.06	−0.01 ± 0.04	0.93 ± 0.04	0.94 ± 0.04	0.9	0.001 ± 0.02	0.5	0.2
% body fat	40.3 ± 8.2	39.9 ± 8.1	0.1	−0.4 ± 1.6	38.8 ± 9.4	38.5 ± 9.2	0.3	−0.3 ± 1.7	0.4	0.9
Fat mass (kg)	36.4 ± 9.8	36.0 ± 9.4	0.3	−0.5 ± 2.1	33.7 ± 10.2	33.4 ± 10.6	0.4	−0.3 ± 2.4	0.3	0.9
Fat free mass (kg)	53.9 ± 12.9	54.2 ± 12.7	0.2	0.3 ± 1.4	53.1 ± 12.2	53.0 ± 11.9	0.3	−0.9 ± 1.5	0.9	0.1
hsCRP (mg/L)	2.6 (1.0, 5.8)	2.0 (1.2, 5.2)	0.9	−0.1 ± 1.4	1.1 (0.9, 3.1)	1.3 (0.6, 2.7)	0.8	−0.4 ± 2.3	0.1	0.9
TNF (pg/ml)	29.6 (14.6, 55.0)	24.3 (11.1, 45.6)	0.3	−8.0 ± 109.4	27.6 (16.9, 72.9)	21.5 (12.4, 47.4)	0.2	−2.9 ± 103.9	0.8	0.9
MCP-1 (pg/ml)	625 (398.4, 951.4)	554.1 (429.6, 925.6)	0.6	−234.8 ± 1071.9	713.0 (483.2, 1087.3)	562.5 (402.5, 851.5)	0.3	−58.8 ± 819.4	0.6	0.6
IFN-α (pg/ml	12.8 (9.8, 27.9)	13.1 (6.5, 23.7)	0.6	11.1 ± 85.8	13.6 (8.4, 28.8)	10.8 (7.4, 22.5)	0.4	−1.3 ± 22.7	0.8	0.9
IL-1β (pg/ml)	15.2 (10.3, 30.1)	14.3 (4.4, 23.2)	0.5	−3.1 ± 39.0	20.1 (9.2, 39.0)	13.8 (6.7, 19.4)	0.3	−4.8 ± 38.1	0.7	0.6
IL-6 (pg/ml)	20.1 (10.0, 30.9)	16.4 (6.0, 35.4)	0.2	−7.7 ± 75.1	21.7 (11.8, 44.4)	14.5 (7.2, 35.2)	0.4	−4.7 ± 65.4	0.6	0.9
IL-8 (pg/ml)	12.7 (7.8, 18.6)	12.2 (7.6, 23.2)	0.8	−2.4 ± 21.9	14.6 (10.2, 24.1)	11.6 (8.8, 17.8)	0.3	−1.2 ± 20.5	0.3	0.7
IL-10 (pg/ml)	8.1 (6.5, 12.9)	7.2 (5.2, 10.4)	0.1	−2.6 ± 20.2	8.7 (6.2, 17.0)	7.4 (5.9, 10.1)	0.3	−0.9 ± 13.3	0.9	0.9
IL-12 (pg/ml)	7.4 (5.4, 9.9)	6.3 (4.6, 8.4)	0.2	−0.8 ± 11.5	6.9 (5.4, 13.0)	5.9 (5.1, 7.6)	0.2	−0.9 ± 10.2	0.9	0.9
IL-18 (pg/ml)	174.7 (101.9, 284.5)	129.1 (87.7, 210.4)	0.2	−100.0 ± 381.9	196.1 (112.1, 265.2)	117.4 (81.2, 211.9)	0.2	−4.7 ± 291.9	0.6	0.9
IL-23 (pg/ml)	25.5 (19.6, 41.3)	17.6 (12.9, 39.3)	0.2	−7.4 ± 126.4	28.6 (16.2, 73.0)	22.4 (9.0, 38.3)	0.2	−10.8 ± 60.5	0.8	0.8
IL-33 (pg/ml)	34.2 (10.4, 118.6)	37.0 (5.4, 79.2)	0.7	−16.2 ± 236.3	48.6 (24.0, 211.0)	23.4 (5.4, 64.4)	0.2	−14.5 ± 228.1	0.4	0.5
NFκB activity in PBMCs (pg/μg protein)	33.6 (26.6, 63.6)	28.6 (23.2, 35.1)	0.05	−17.8 ± 31.9	38.2 (23.1, 67.1)	27.7 (23.0, 34.8)	0.2	−13.2 ± 32.4	0.8	0.7

Data are expressed as mean ± standard deviation, or median (interquartile range) for non-normally distributed variables, unless otherwise specified. Analyses adjusted for multiple testing using Bonferroni correction, such that *P* < 0.004 was considered statistically significant.

*p*_1_ = Wilcoxon sign rank tests for differences between baseline and follow up within vitamin D group;

*p*_2_ = Wilcoxon sign rank tests for differences between baseline and follow up within placebo group;

*p*_3_ = Mann-Whitney U-tests for differences at baseline between vitamin D and placebo groups;

*p*_4_ = Mann-Whitney U-tests for differences in change scores between vitamin D and placebo groups.

Abbreviations: 25(OH)D, 25-hydroxyvitamin D; BMI, body mass index; WHR, waist-to-hip ratio; hsCRP, high-sensitivity C-reactive protein; TNF, tumor necrosis factor; MCP-1, monocyte chemoattractant protein-1; IL, interleukin; NFκB, nuclear factor *kappa*-B; PBMC, peripheral blood mononuclear cells.

### Effect of vitamin D supplementation on serum pro- and anti- inflammatory markers and NFκB activity in PBMCs

There were no differences between vitamin D and placebo groups in change in hsCRP (−0.1 ± 1.4 versus −0.4 ± 2.3 mg/L, p = 0.9), TNF (−8.0 ± 109.4 versus −2.9 ± 103.9 pg/ml, p = 0.9), MCP-1 (−234.8 ± 1071.9 versus −58.8 ± 819.4 pg/ml, p = 0.6) or IFN-α (11.1 ± 85.8 versus −1.3 ± 22.7 pg/ml, p = 0.9). Similarly, no differences between vitamin D and placebo groups were found for any of the interleukins (all p > 0.1; Table 2). Changes in pro-/anti-inflammatory marker ratios including hsCRP/IL-10, TNF/IL-10 and IL-6/IL-10 ratios also did not differ between groups (data not shown). Change in NFκB activity in PBMCs did not differ between vitamin D and placebo groups (−17.8 ± 31.9 versus −13.2 ± 32.4 pg/μg protein, p = 0.7; Table 2); however change in NFκB activity within the vitamin D group (between baseline and follow up) trended toward significance (p = 0.05; Table 2).

Results for all inflammatory markers and NFκB activity remained non-significant in multiple regression analyses adjusted for baseline values, age, sex, ethnicity, and season of blood collection (all p > 0.1; Table 3, Model 1). Replacing ethnicity and season of blood collection in the multivariable model with factors which may affect vitamin D status including dietary vitamin D intake, sun exposure, and physical activity did not alter the results (all p > 0.1; Table 3, Model 2). Results remained non-significant in a third model adjusting for baseline values and factors which are clinically relevant to both vitamin D status and inflammation including change in % body fat, dietary vitamin D intake, diet composition (as fat/carbohydrate ratio), physical activity, and sun exposure (all p > 0.1; Table 3, Model 3). Addition of protein and/or fiber intake to the models as well as replacing % body fat with fat mass only or with BMI and WHR did not change the results (data not shown).

**Table 3:** Multivariable regression analysis for differences in outcomes between the vitamin D group and placebo group (reference group) after adjustment for covariates.

Dependent Variable (Change)	Model 1			Model 2			Model 3		
	*β*	*SE*	*p*	*β*	*SE*	*p*	*β*	*SE*	*p*
hsCRP (mg/L)	0.24	0.54	0.7	−0.15	0.45	0.7	−0.29	0.58	0.6
TNF (pg/ml)	3.41	10.91	0.8	1.42	12.63	0.9	0.15	11.44	0.9
MCP-1 (pg/ml)	−53.64	169.50	0.8	4.26	177.93	0.9	−5.45	241.60	0.9
IFN-α (pg/ml)	0.27	7.54	0.9	0.24	6.19	0.9	1.00	6.21	0.9
IL-1β (pg/ml)	4.27	5.54	0.4	1.57	6.36	0.8	0.61	6.83	0.9
IL-6 (pg/ml)	−2.37	8.19	0.8	0.94	9.98	0.9	0.50	9.08	0.9
IL-8 (pg/ml)	0.18	4.08	0.9	2.08	4.79	0.7	−0.37	4.71	0.9
IL-10 (pg/ml)	−0.58	1.69	0.7	−0.47	2.41	0.8	0.81	2.96	0.8
IL-12 (pg/ml)	0.58	1.24	0.6	−0.23	1.24	0.9	−0.75	1.48	0.6
IL-18 (pg/ml)	−1.48	45.48	0.9	−2.62	48.03	0.9	−43.09	45.50	0.4
IL-23 (pg/ml)	−1.43	9.23	0.9	−7.64	12.92	0.6	−4.51	17.60	0.8
IL-33 (pg/ml)	2.31	28.88	0.9	15.10	39.12	0.7	13.70	41.69	0.7
NFκB activity (pg/μg protein)	−0.25	4.05	0.9	2.23	3.15	0.5	4.48	3.70	0.2

Data was analyzed using quantile (median) regression for differences in median change scores between groups after adjusting for covariates (placebo group as reference group). Results presented as unstandardized beta coefficients (β), standard error (SE), and corresponding *p*-values. All analyses were adjusted for multiple testing using Bonferroni correction, such that *p* < 0.004 was considered statistically significant. **Model 1:** adjusted for baseline values, age, sex, ethnicity, and season of blood collection; **Model 2:** adjusted for baseline values, age, sex, change in dietary vitamin D intake, physical activity (international physical activity questionnaire-multiples of the resting metabolic rate), and sun exposure index; **Model 3:** adjusted for baseline values, % body fat, dietary vitamin D intake, diet composition (fat/carbohydrate ratio), physical activity (international physical activity questionnaire-multiples of the resting metabolic rate), and sun exposure index. Abbreviations: hsCRP, high-sensitivity C-reactive protein; TNF, tumor necrosis factor; MCP-1, monocyte chemoattractant protein-1; IL, interleukin.

### Sub-group Analyses

Results of the subgroup analyses are presented in Table 4. Pre-specified subgroup analysis was conducted in participants with 25(OH)D concentrations ≤30 nmol/L (n = 23; 15 males/8 females; age = 32.0 ± 7.6 years). In this subgroup, baseline 25(OH)D concentration was 21.7 ± 5.7 nmol/L (range = 9–29 nmol/L), with a mean BMI of 30.5 ± 4.2 kg/m^2^ and % body fat of 40.3 ± 8.7%. Baseline demographic, anthropometric, and biochemical characteristics did not differ between groups (data not shown). Serum 25(OH)D concentrations increased in the vitamin D group compared to placebo (59.9 ± 20.2 versus 6.9 ± 18.4 nmol/L, p < 0.001). There were no significant differences between vitamin D and placebo groups in any of the pro- or anti-inflammatory markers measured (all p > 0.1; Table 4). NFκB activity also did not differ between vitamin D and placebo groups in this subgroup (p = 0.3; Table 4).

**Table 4:** Subgroup analyses of participants with 25-hydroxyvitamin D concentrations ≤ 30 nmol/L and participants with a body mass index ≥ 30 kg/m^2^.

Outcome Variable (Change scores)	25(OH)D < 30 nmo/L Subgroup (n = 23)			BMI > 30 kg/m^2^ Subgroup (n = 28)		
	Vitamin D Group (n = 14)	Placebo Group (n = 9)	*p*	Vitamin D Group (n = 13)	Placebo Group (n = 15)	*p*
25(OH)D (nmol/L)	59.9 ± 20.2	6.9 ± 18.4	<0.001	48.5 ± 19.7	−2.1 ± 12.1	<0.001
hsCRP (mg/L)	−0.1 ± 1.4	−1.5 ± 3.3	0.5	0.3 ± 1.3	0.3 ± 1.6	0.8
TNF (pg/ml)	3.5 ± 146.9	−27.1 ± 49.6	0.3	−24.4 ± 82.5	−18.6 ± 60.1	0.9
MCP-1 (pg/ml)	−291.9 ± 1404.8	−86.4 ± 712.2	0.6	−320.8 ± 1319.3	−235.8 ± 529.9	0.3
IFN-α (pg/ml)	28.7 ± 119.7	−5.4 ± 17.4	0.4	−5.9 ± 22.0	−3.9 ± 8.4	0.7
IL-1β (pg/ml)	6.4 ± 50.3	−10.4 ± 22.7	0.1	−10.5 ± 24.9	−11.4 ± 21.2	0.7
IL-6 (pg/ml)	7.1 ± 91.2	−21.8 ± 42.4	0.5	−23.8 ± 60.4	−14.2 ± 42.1	0.8
IL-8 (pg/ml)	0.9 ± 30.3	−4.8 ± 12.7	0.2	−6.0 ± 22.3	−5.4 ± 10.4	0.5
IL-10 (pg/ml)	−1.6 ± 28.4	−4.6 ± 8.3	0.5	−4.0 ± 9.5	−3.2 ± 8.9	0.7
IL-12 (pg/ml)	1.0 ± 15.8	−3.2 ± 5.7	0.3	−3.0 ± 7.4	−2.2 ± 5.5	0.9
IL-18 (pg/ml)	−135.9 ± 530.5	−99.5 ± 203.6	0.7	−59.8 ± 220.8	−57.6 ± 169.0	0.4
IL-23 (pg/ml)	25.8 ± 140.0	−23.5 ± 44.6	0.3	−37.6 ± 109.0	−21.5 ± 38.9	0.7
IL-33 (pg/ml)	17.1 ± 315.9	−49.7 ± 97.4	0.1	−55.5 ± 172.3	−47.3 ± 114.4	0.6
NFκB activity (pg/μg protein)	−11.7 ± 37.9	5.8 ± 19.3	0.4	−26.7 ± 25.7	−17.2 ± 37.8	0.3

Data are expressed as mean ± standard deviation for change scores. All analyses performed using Mann-Whitney U-tests for differences between groups, and adjusted for multiple testing using Bonferroni correction, such that *p* < 0.004 was considered statistically significant. Abbreviations: 25(OH)D, 25-hydroxyvitamin D; hsCRP, high-sensitivity C-reactive protein; TNF, tumor necrosis factor; MCP-1, monocyte chemoattractant protein-1; IL, interleukin.

Subgroup analysis was also conducted to explore the effects of vitamin D supplementation in obese participants (BMI ≥ 30 kg/m^2^) (n = 28; 13 males/15 females; age = 33.7 ± 9.0 years). Mean baseline 25(OH)D concentration in this subgroup was 34.6 ± 11.1 nmol/L (range = 9–50 nmol/L), with a BMI of 34.1 ± 3.8 kg/m^2^ and % body fat of 44.2 ± 8.4%. There were no differences between groups in demographic, anthropometric or biochemical characteristics at baseline. Changes in pro- and anti-inflammatory markers and NFκB activity did not differ between vitamin D and placebo groups in this subgroup of obese participants (Table 4). Further exploratory analyses were conducted to examine the effects of vitamin D supplementation in subgroups of participants with high baseline concentrations (≥median values) of selected inflammatory markers and NFκB activity. Changes in inflammatory markers and NFκB activity did not differ between vitamin D and placebo groups in subgroups of participants with high CRP (≥2.0 mg/L), IL-6 (≥21.0 pg/ml), TNF (≥28.0 pg/ml) or NFκB activity (≥37.0 pg/μg protein) (data not shown). Finally, we compared those in the vitamin D group who reached a 25(OH)D concentration >70 nmol/L (n = 23) or >75 nmol/L (n = 18) at follow up with the placebo group (n = 23 with 25(OH)D < 50 nmol/L at follow up) and found no differences in any of the parameters measured (all p > 0.05; data not shown).

## Discussion

This randomized placebo-controlled trial examined the effect of oral cholecalciferol (100,000 IU bolus followed by 4,000 IU daily) supplementation for 16 weeks on pro- and anti-inflammatory markers and NFκB activity in overweight or obese but otherwise healthy individuals with vitamin D deficiency (25(OH)D ≤ 50 nmol/L). Despite a significant increase in 25(OH)D concentration in the vitamin D group, we found no differences between vitamin D and placebo groups in any of the pro- or anti- inflammatory markers measured, including hsCRP, TNF, MCP-1, IFN-α, and IL-1β, -6, -8, -10, -12, -18, -23, and -33, as well as no difference in NFκB activity in PBMCs. Adjustment for potential confounders did not alter the results and similar results were found in subgroup analyses of individuals with 25(OH)D ≤ 30 nmol/L, obese individuals (BMI > 30 kg/m^2^), or individuals with higher inflammatory marker or NFκB concentrations at baseline (concentrations ≥ median for hsCRP, TNF, IL-6, and NFκB activity).

Previous RCTs in overweight and obese but otherwise healthy adults have reported similar findings. A recent meta-analysis of 13 RCTs^26^ including 1,995 overweight and obese participants reported that vitamin D supplementation had no effect on plasma CRP, TNF, or IL-6 concentrations. With the exception of three trials^33–35^, all the included RCTs reported no effect of vitamin D supplementation on several pro- and anti-inflammatory markers including but not limited to hsCRP, TNF, MCP-1, and several interleukins (IL-2, -4, -5, -6, -8, -10, -12, -13, and -17)^26^. Of the three studies which found a beneficial effect, one had co-supplemented vitamin D with calcium^33^, and another combined vitamin D with a weight reduction program^35^. This makes results difficult to interpret since both calcium and weight loss have been shown to influence levels of circulating inflammatory markers^36,37^. The third study reporting that vitamin D supplementation improved inflammatory markers was limited to South American women^34^. This may explain their discrepant finding since polymorphisms in the VDR and metabolizing enzymes which affect the absorption and genetic functions of vitamin D are known to differ by ethnicity^34,38^.

Overall, existing RCTs in overweight and obese populations are limited by several important factors. First, most RCTs did not take into account participant smoking status, season, sun exposure, or dietary vitamin D intake – factors which may have influenced their results^39,40^. Second, 80% of existing RCTs were not in vitamin D-deficient individuals and this group has been proposed to benefit most from vitamin D supplementation^41^. Although we found no effect in our subgroup analysis of participants with 25(OH)D concentrations ≤ 30 nmol/L, this may be due to the small sample size. Importantly, the two largest RCTs^33,42^ (n = 928 and n = 332) in overweight and obese adults were in participants without vitamin D deficiency (baseline 25(OH)D range = 56–136 nmol/L), and both trials reported increased CRP concentrations after one year of supplementation with 20,000–40,000 IU of cholecalciferol weekly^33,42^. This suggests that high doses of vitamin D in non-deficient participants may be detrimental^43^, and highlights the need for similar large-scale studies that are restricted to individuals with vitamin D deficiency. Third, it has been suggested that at least 4,000 IU daily is required to raise 25(OH)D concentrations to optimal levels within 2–3 months^44,45^. Yet, more than half of the RCTs supplemented low doses (700–2000 IU daily) which may have been insufficient to adequately raise 25(OH)D concentrations. Here, we address these knowledge gaps by showing that vitamin D supplementation, provided in a sufficient dose to vitamin D-deficient individuals, did not improve circulating pro- or anti-inflammatory marker concentrations, which persisted after adjusting for multiple confounders. Based on our findings and those from most other studies, current evidence does not support the use of vitamin D supplementation for improving inflammation in overweight or obese, but otherwise healthy individuals.

However, vitamin D supplementation may be beneficial in other population groups including those with existing chronic or inflammatory diseases. RCTs and systematic reviews of RCTs have shown that vitamin D supplementation improved inflammatory marker concentrations in patients with chronic heart failure^46^, systemic lupus erythematosus^47^, inflammatory bowel disease^48^, and chronic obstructive pulmonary disease^49^. Moreover, although RCTs in prediabetes have shown no effect of vitamin D supplementation on inflammatory markers^50–52^, a meta- analysis by our group in patients with type 2 diabetes found that vitamin D-supplemented groups had lower levels of CRP and TNF compared to placebo (Mousa, unpublished data). It has been suggested that the effects of vitamin D on the immune system are more prominent when the immune system is impaired or stimulated^53^. Differences between studies may therefore be due to the presence of acute or chronic inflammation in participants with established diseases compared to their healthy counterparts. This is supported by a meta-analysis of mixed population groups (healthy and with existing diseases; n = 924)^54^, which found that vitamin D supplementation had a significantly greater effect on inflammation in patients with higher CRP levels (>5 mg/L) at baseline, compared to normal ranges. Subgroup analysis of participants with CRP levels >5 mg/L was not possible in our study due to the small number of participants with elevated CRP (n = 11). Moreover, although we found no differences between vitamin D and placebo groups in our subgroup analyses of participants with baseline concentrations > the median for CRP, TNF, IL-6, and NFκB activity, our sample size may have been too small to observe effects in these subgroups (n = 27). Future studies in healthy individuals should consider including large samples of vitamin D-deficient participants with a wide range of inflammatory marker concentrations at baseline in order to confirm whether vitamin D supplementation improves pro- and anti- inflammatory profiles in this population group.

We found no effect of vitamin D supplementation on NFκB activity in PBMCs. To the best of our knowledge, this is the first study to investigate the effects of vitamin D supplementation on NFκB activity *in vivo* in humans. Only experimental human studies and animal models have investigated this relationship to date, most of which reported that vitamin D inhibits NFκB activity in several human cell types including macrophages, fibroblasts, keratinocytes, pancreatic islet cells, and adipocytes^10–14^. These *in vitro* studies have consistently shown that treatment with active vitamin D (1,25(OH)_2_D_3_) increases mRNA and protein levels of IκBα (an inhibitor of nuclear translocation of NFκB), which in turn decreases NFκB activity and DNA-binding capacity, and reduces nuclear translocation of the NFκB p65 subunit^10–14^. However, some studies showed that 1,25(OH)_2_D_3_ can both stimulate and inhibit NFκB in different types of myeloid leukemic cells^55,56^. This suggests that vitamin D may influence NFκB activity in a cell- and tissue- specific manner. Similarly, *in vivo* animal models have shown that the VDR directly regulates the NFκB pathway^57^, and that treatment with active 1,25(OH)_2_D_3_ or analogs (eg: paricalcitol) increases VDR expression, with concomitant reductions in the NFκB signaling cascade^18,19^. Our finding of no effect of vitamin D supplementation on NFκB activity in PBMCs seems to conflict with the widely reported inhibitory effect of vitamin D in experimental and animal models. However, experimental studies have explored the active form of vitamin D_,_ 1,25(OH)_2_D_3_, and have utilized different stimuli including bacteria, viruses, or agents like lipopolysaccharide (LPS) that activate transcription factors including NFκB^58^. Because our study measured 25(OH)D rather than 1,25(OH)_2_D_3_, and NFκB activity was measured in unstimulated PBMCs, these factors may partly explain the discrepancy between our findings and those from experimental studies. Moreover, vitamin D and NFκB may function differently in the natural biological environment in humans (ie: *in vivo*) compared to what is observed in animals or under experimental conditions. For instance, a study by Wamberg *et al*.^14^ in obese individuals found that 1,25(OH)_2_D_3_ treatment inhibited cytokine expression *in vitro*; however no effect was observed after *in vivo* treatment with 1,25(OH)_2_D_3_ in the same population. This suggests that in some circumstances, extrapolation of *in vitro* findings to the *in vivo* context may not be appropriate. Here, we show for the first time that vitamin D supplementation has no effect on *in vivo* NFκB activity in vitamin-deficient and overweight or obese but otherwise healthy individuals. Further studies are needed to corroborate our finding and to examine whether these effects may differ in other cell types or among other populations including those with existing inflammatory or chronic diseases.

Our study has several strengths, particularly the use of rigorous methodology and a double-blind randomized controlled design. This was the first study investigating the effects of vitamin D supplementation on NFκB activity *in vivo* in humans. We studied overweight or obese participants with vitamin D deficiency who were more likely to be at increased risk of inflammation and who have been proposed to benefit most from vitamin D supplementation. Compliance to study medication was high, with 82% of participants in the intervention group achieving 25(OH)D concentrations >70 nmol/L at follow-up. Our study sample comprised of a well-characterized cohort of healthy individuals where there was no confounding by disease status or medication use. We were able to adjust for important confounders including season, sun exposure, and dietary vitamin D intake- factors which have seldom been considered in previous studies.

Some limitations should be noted. Pro- and anti- inflammatory markers and NFκB activity were secondary outcomes of the main trial where the sample size calculation was based on changes in insulin sensitivity^27^. Our sample size was therefore not powered to detect differences in these outcomes or to draw valid conclusions from subgroup analyses. Moreover, most participants had hsCRP levels within a normal range (<5 mg/L) and narrow ranges for most of the other inflammatory markers. This may have contributed to our null findings since any changes in inflammatory markers would have been minimal, thereby requiring a much larger sample size in order to detect small effect sizes. Additionally, although 82% of participants in the vitamin D group reached a serum 25(OH)D level >70 nmol/L at follow up, it has been suggested that 25(OH)D concentrations as high as 80–100 nmol/L may be required for optimal immune function^59^, which may explain our lack of findings. Because our sample comprised of healthy young individuals, our results may not be generalizable to other populations including those with existing diseases. We measured NFκB activity in PBMCs, thus our results may not apply to other cell or tissue types. Potential contributions by genetic polymorphisms in the VDR or metabolizing enzymes were not explored in this study. Although *in vitro* data suggest that the biologically active vitamin D (1,25(OH)_2_D_3_) is involved in immunoregulation, we did not measure this form of vitamin D because circulating levels are tightly regulated^60^. Due to resource constraints, we were not able to measure 25(OH)D using the gold-standard liquid chromatography-mass spectrometry method, and instead used Diasorin assays which capture both 25(OH)D_2_ and 25(OH)D_3_ concentrations.

In summary, the present study addresses current knowledge gaps by providing high-doses of vitamin D supplementation to vitamin D-deficient individuals, without co-interventions, and with adjustment for important confounders – all of which are factors that have not been adequately addressed in previous trials. We found no beneficial effect of vitamin D supplementation on circulating pro- and anti-inflammatory markers or NFκB activity in overweight or obese but otherwise healthy individuals. Our results suggest that vitamin D may function differently in the human *in vivo* context compared to the inhibitory effects reported in *in vitro* and animal models. However, further trials in healthy individuals are needed to corroborate our findings, with the inclusion of: (1) large sample sizes with sufficient power to detect small changes; (2) participants with vitamin D-deficiency and a wider range of baseline inflammatory marker concentrations; and (3) assessment of whether vitamin D may affect *in vivo* NFκB activity in other cell and tissue types.

## Electronic supplementary material

CONSORT Checklist
